# Detrimental Effects of Workplace Bullying: Impediment of Self-Management Competence via Psychological Distress

**DOI:** 10.3389/fpsyg.2016.00060

**Published:** 2016-02-15

**Authors:** Gabriele Giorgi, Milda Perminienė, Francesco Montani, Javier Fiz-Perez, Nicola Mucci, Giulio Arcangeli

**Affiliations:** ^1^Department of Psychology, European University of RomeRome, Italy; ^2^Department of Philosophy and Psychology, Kaunas Technology UniversityKaunas, Lithuania; ^3^Montpellier Business School, MontpellierFrance; ^4^Institute of Public Health, Catholic University of the Sacred HeartRome, Italy; ^5^Department of Clinical and Experimental Medicine, University of FlorenceFlorence, Italy

**Keywords:** workplace bullying, emotional intelligence, ability of self-management, psychological distress, work-related stress, occupational safety, occupational health

## Abstract

Emotional intelligence has been linked to various positive outcomes, such as organizational effectiveness, commitment, morale, and health. In addition, longitudinal studies demonstrate that the competencies of emotional intelligence may change and be developed over time. Researchers have argued that work relationships are important for the development of emotional competence, but their usefulness depends on the quality of the relationship. Workplace bullying is considered to be one of the most stressful phenomena in the workplace and an example of a dysfunctional and toxic relationship that has detrimental effects on an individual’s physical and psychological health. Hence, the objective of the present study was to analyze the relationship linking workplace bullying, psychological distress and the self-management competence of emotional intelligence. More specifically, we tested part of the model presented by Cherniss and Goleman (2001) in which researchers argued that individual emotional intelligence is a result of relationships at work. In addition, we extended the model by proposing that the relationship between exposure to workplace bullying and the competence of self-management is explained by psychological distress. Data analysis of 326 participants from two private sector organizations in Italy demonstrated that psychological distress fully mediated the relationship between workplace bullying and the emotional intelligence ability of self-management. The present study’s findings point to the idea that, not only may emotional intelligence assist in handling exposure to workplace bullying, but exposure to workplace bullying may impede emotional intelligence via psychological distress.

## Introduction

According to Einarsen et al. (2011, p. 15), “Bullying at work means harassing, offending, socially excluding someone, or negatively affecting someone’s work tasks." It is a gradual process in which an individual is subjected to indirect and subtle forms of psychological violence (also referred to as negative acts; Einarsen et al., 2011). The negative behaviors are repeated in a systematic way (e.g., on a weekly or daily basis) and over a prolonged period of time (e.g., at least 6 months; Einarsen et al., 2011). An individual exposed to bullying behaviors may end up leaving the organization and/or suffering severe psychological trauma (Leymann, 1996; Einarsen et al., 2011).

A considerable number of studies have focused their attention on the mental and physical health consequences of workplace bullying. Empirical investigations in a wide variety of countries provide data that point to the negative consequences of bullying. Results of a study in the United States demonstrated that mistreated workers presented poorer self-evaluations on their health status and a perceived workplace mistreatment was related to a 42% increase in the expected number of days of absence from work (Asfaw et al., 2014). A study among Turkish employees demonstrated that bullied workers reported lower levels of job satisfaction, higher levels of job-induced stress and higher anxiety and depression scores (Bilgel et al., 2006). A study in the Netherlands showed that employees reporting weekly bullying experienced more health problems, poorer well-being, and were more frequently absent from work (Dehue et al., 2012). Among working students in Australia, exposure to workplace bullying was linked to physical symptoms, negative effect, and intentions to leave their job (Djurkovic et al., 2004).

In addition, workplace bullying is one of the major factors that increases costs for organizations (especially in a small or medium business, among young and older workers, disabled workers, particular industry sectors, poor work organization, etc.; Arcangeli and Mucci, 2009; Giorgi et al., 2014c; Mucci et al., 2015a).

The increased costs for organizations are due to employee turnover and absenteeism (Hauge et al., 2010; Arcangeli et al., 2014; Giorgi et al., 2015a), lower work motivation (Pranjić et al., 2006), reduced productivity and commitment (Pearson et al., 2000), and interventions by health officers and personnel managers (Leymann, 1990).

Numerous studies have highlighted the significant associations between individual (personality) factors and the exposure to workplace bullying. A Norwegian study, conducted in a sample of 2200 workers, brought to light that the victims of bullying were characterized by low self-esteem and social competence, and reported high levels of anxiety (Einarsen et al., 1994). Coyne et al. (2000), in a study of 60 Irish victims of bullying, found that bullied individuals were: (1) more anxious and suspicious, (2) less assertive, and (3) had limited emotional coping resources. Perminiene et al. (2016) demonstrated that more rule-focused, more bossy and controlling, and more cautious individuals are exposed to greater workplace bullying. On the other hand, Leymann (1996) disregarded the view that individual characteristics of targets may be antecedents of workplace bullying and, instead, claimed that an individual may experience major personality changes as a consequence of exposure to workplace bullying. Indeed, recent literature points out that emotional intelligence might decrease with the occurrence of workplace bullying (Giorgi and Majer, 2009). In line with that, Kram and Cherniss (2001) proposed that future research related to emotional intelligence should address how peer relationships foster (or impede) the development of particular abilities and competencies of emotional intelligence. Hence, in the present study, we aim to identify how toxic peer relationships, i.e., workplace bullying, are linked to the ability of emotional intelligence (self-management).

Although Cherniss and Goleman (2001) argued that relationships at work may relate to the development of emotional intelligence, such a narrow model may be incomplete. Researchers have indicated that other mechanisms may be considered in future studies (Cherniss and Goleman, 2001). Aiming at expanding and, hence, clarifying the model, we also aim to test whether psychological distress explains the indirect relationship between exposure to workplace bullying and the ability of emotional intelligence (self-management). Hence, the overall objective of the present study is to analyze the relationship linking workplace bullying, psychological distress and the self-management ability of emotional intelligence.

### Emotional Intelligence and Workplace Bullying

Emotional intelligence is the ability to perceive, express and understand emotions and to be able to regulate them in ourselves and in others (Salovey and Mayer, 1990; Mayer and Salovey, 1997; Cabello and Fernández-Berrocal, 2015). There are several different models that aim to explain the construct of emotional intelligence (Cherniss et al., 2006). According to some researchers, emotional intelligence is a relatively stable construct. For example, Petrides et al. (2007) identified trait emotional intelligence and demonstrated that it is a distinct, compound (partially determined by several personality dimensions) construct that lies at the lower levels of personality hierarchies. On the other hand, according to Cherniss et al. (2006), emotional intelligence is distinct from IQ and the Big Five personality traits. In addition, researchers have highlighted that it includes a variety of competencies and skills that can be developed during a lifespan (Goleman, 1998). According to Boyatzis (2001), longitudinal studies at the Weatherhead School of Management (WSOM) have shown that, over 2–5 years, people can develop and change the competencies of emotional intelligence.

Understanding what encourages and what impedes the development of emotional intelligence is important, because its development seems to be linked to the success of an individual as well as an organization. According to Cherniss and Goleman (2001), emotionally intelligent leaders and employees contribute to organizational effectiveness, quality of service, good employee recruitment, retention, commitment, morale, and health. According to Yadav (2014), emotionally intelligent employees and leaders are cheerful, inculcate a sense of enthusiasm, positive attitude, excitement and an atmosphere of mutual understanding and trust. Several empirical studies have demonstrated the positive outcomes of emotional intelligence for individuals. For example, Rosete and Ciarrochi (2005) found a link between emotional intelligence and leadership effectiveness. A study by Cavallo and Brienza (2004) showed that superior performers scored higher on emotional intelligence competencies (e.g., self-awareness, self-management, social awareness, and relationship management). Mortan et al. (2014) demonstrated that dimensions of emotional intelligence are linked to self-efficacy. Despite a number of studies demonstrating the positive outcomes of emotional intelligence, there is little research aimed at identifying what potentially encourages or impedes the development of the abilities of emotional intelligence.

In Goleman’s (2001) framework of emotional competencies, emotional intelligence was described through the four key abilities of self-awareness, self-management, social awareness, and relationship management. These four abilities may be detailed further into specific competencies. In **Table 1**, each of the four key abilities is described by the more specific competencies of emotional intelligence.

**Table 1:** Fit indices for confirmatory factor analyses.

Model	χ^2^	*df*	Δχ^2^	Δ*df*	CFI	RMSEA	SRMR
Hypothesized three-factor model	37.710^∗^	11	–	–	0.95	0.09	0.04
Two-factor model (combining psychological distress and ability of self-management)	54.209^∗∗^	13	16.499^∗∗^	2	0.91	0.10	0.05
One-factor model	88.936^∗∗^	14	51.226^∗∗^	3	0.84	0.13	0.06

*N = 326. CFI, comparative fit index; RMSEA, root-mean-square error of approximation; SRMR, standardized root mean square residual; ∗p < 0.05, ^∗∗^p <0.01.*

One of the abilities of emotional intelligence, e.g., the self-management ability, was described as an ability to manage one’s internal impulses and resources, keeping disruptive emotions and impulses in check, maintaining standards of honesty and integrity, taking responsibility for personal performance, flexibility in handling change, and being comfortable with novel ideas, approaches, and new information (Goleman, 1998). Originally, Goleman conceptualized the cluster of self-management as two clusters of self-regulation and motivation. The self-regulation cluster involved managing and controlling one’s impulses, whereas motivation involved energizing and driving individual’s behavior (Jacobs, 2001).

Self-management is important to understand, because it was found to be one of the key abilities of emotional intelligence. For example, Giorgi et al. (2014a) demonstrated that self-management predicted sales’ success. Researchers argue that individuals who display good self-management are more likely to engage in their work with clients with a relaxed and organized approach and they know which emotions to display (Giorgi et al., 2014a). In addition, Yadav (2014) claimed that an individual who can manage themselves can also handle relationships, and achieve personal and professional goals, leading to success. Hence, understanding the potential causes of self-management development is critical for the benefit of both an individual and an organization.

Kram and Cherniss (2001) suggested some ideas on how abilities and competencies of emotional intelligence develop. They argued that work relationships are important for the development of emotional competencies, but their usefulness depends on the quality of the relationship. According to research, some relationships may even be destructive in regards to the development of emotional competencies (Kram and Cherniss, 2001).

One of the most dysfunctional phenomena in the workplace is workplace bullying (Fox and Stallworth, 2010; Hauge et al., 2010). There are several arguments suggesting a potential link between the exposure to workplace bullying and self-management ability. For example, research on the targets of bullying indicated that employees who have been exposed to bullying at work were oversensitive, suspicious, blamed others and were more resentful and angry (Gandolfo, 1995). They also lacked social competence (Matthiesen and Einarsen, 2001, 2004), were less social and talkative, and were less likeable, understanding, and diplomatic (Glasø et al., 2007; Lind et al., 2009). Persson et al. (2009) demonstrated that bullied individuals displayed higher irritability and impulsiveness scores. All these characteristics seem to point to the lack of competencies of self-management ability (Goleman, 2001). In addition, Glasø et al. (2007) demonstrated that some victims of workplace bullying scored lower on conscientiousness, which is considered to be part of self-management ability.

Baumeister et al. (2005) claimed that because self-regulation (similar to self-management) exists partly for the sake of securing and maintaining social acceptance, it may be affected by social rejection. In six experiments, they indeed demonstrated that being excluded or rejected caused decrements in self-regulation (Baumeister et al., 2005). Previous research also demonstrated that social rejection is linked to a drop in cognitive functioning, lower resistance to temptations and limited capacities of proper social functioning (Baumeister et al., 2005). DeWall et al. (2007), in five experimental studies, found that previous efforts at self-regulation weakened inner restraints and increased the chances of aggressive impulses and aggressive behavior. Considering that workplace bullying requires effort in self-regulation, one may hypothesize that, in the long-term, it could lead to depleted self-regulation and low impulse control and, hence, to impeded self-management ability. Adams and Webster (2013) found that interpersonal mistreatment was related to emotional regulation, which is similar to the construct of self-management. Based on theoretical and empirical arguments, we propose the first hypothesis:

*Hypothesis 1*: Exposure to workplace bullying is negatively related to self-management ability.

### Workplace Bullying, Self-Management, and Psychological Distress

Empirical evidence on workplace bullying has presented a wide range of a wide range of tangible (financial losses, reduced productivity) and intangible costs (interpersonal relationships, mood disorders) for individuals and organizations. Various researchers throughout the world have reported severe consequences, such as: stress (Arcangeli et al., 2014; Giorgi et al., 2014b,c 2015b; Mucci et al., 2015b); psychosomatic symptoms (Mikkelsen and Einarsen, 2001; Hansen et al., 2010); anxiety (Leymann, 1990, 1996); depression (Björkqvist et al., 1994; Hansen et al., 2006); fatigue and loss of self-confidence (Vartia, 1996; Pranjić et al., 2006), aggression, insomnia, and apathy (Björkqvist et al., 1994); and muscle pains, headaches, stomach problems, anxiety attacks, and hand tremors (Celep and Konakli, 2013). Giorgi et al. (2015c) found that workplace bullying was related to poor psychological health, which was, in turn, linked to dysfunctional behaviors. The seriousness of the phenomenon may be supported by the fact that workplace bullying was identified as the strongest predictor of anxiety and depression when compared to other job-related stressors (Hauge et al., 2010). A link between workplace bullying and depression has been established in longitudinal research (Figueiredo-Ferraz et al., 2015). In addition, it was claimed that, in the most severe cases, individuals may commit suicide due to unbearable experiences (Leymann, 1990) or face detrimental consequences such as post-traumatic stress disorder (Leymann and Gustafsson, 1996).

Some of the symptoms of post-traumatic stress disorder are avoidance, social withdrawal, emotional numbing, irritable and angry behavior, and concentration difficulties (Balducci et al., 2009). These symptoms seem to demonstrate that the competencies (such as emotional self-control, conscientiousness, adaptability, and achievement drive) of self-management ability are also impeded. Hence, it seems that the exposure to bullying behaviors may develop into psychological distress, which further leads to the decrease of self-management ability.

The indirect relationship between exposure to workplace bullying and self-management ability (via psychological distress) may also be based on the research on burnout and workplace bullying. For example, Einarsen et al. (1998) demonstrated that bullied assistant nurses had higher levels of burnout and poorer psychological well-being. Part of the peculiarities of the behavior typical for individuals who experience burnout is that these individuals lack adaptability, achievement drive, and initiative (Maslach et al., 2001), which are also parts of the self-management ability.

In addition, previous research has established links between psychological distress and the competencies of self-management ability. For example, Wienke Totura et al. (2014) demonstrated that psychological distress explained the relationship between peer victimization and achievement, which is one of the elements of self-management ability.

There is also a physiological argument on how exposure to workplace bullying may lower self-management ability via psychological distress. Biologically, stressful experiences impair the functions of the prefrontal cortex (which is responsible for flexible, goal-directed behavior) and strengthens the primitive emotional responses of the amygdala (which is primarily responsible for emotional reactions); hence, an ability to inhibit inappropriate impulses, attention regulation, and accurate insights about one’s actions are also impeded (Arnsten et al., 2015). Psychological distress develops as a result of stressful experiences, such as workplace bullying (Finne et al., 2011), which then leads to increased irritability, impaired decision-making, lack of insight (Arnsten et al., 2015). Hence, based on the theoretical and empirical arguments, we designed the second hypothesis:

*Hypothesis 2*: Exposure to workplace bullying is negatively related to self-management ability via increased levels of psychological distress.

**FIGURE 1:**
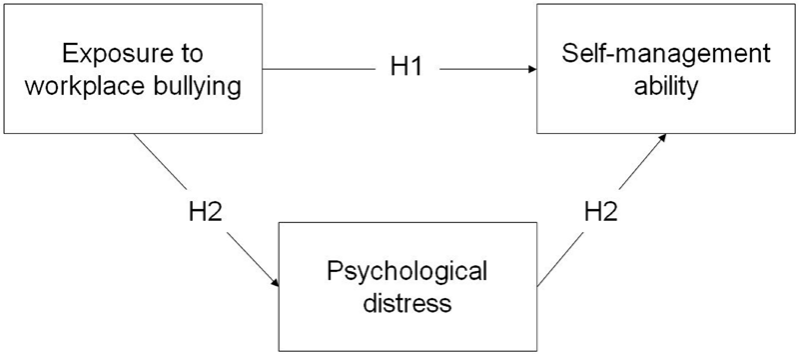
**Hypothesized model of the present study**.

The hypothesized model of the present study is depicted in **Figure 1.**

## Materials and Methods

Written consent was obtained from each participant for the anonymous use of their responses. The Ethics Committees of University of Florence and European University of Rome approved the study.

Data were collected in 2014 and 2015 by means of paper and pencil questionnaires among employees from two Italian companies. The organizations were selected using convenience sample procedure. The first company was a manufacturing organization in the luxury sector (response rate = 80%). Main jobs involved the production of shoes and leather bags. The entire company population (*n* = 172) was invited to participate in the research project. The returned questionnaires were 138. The second company was a service organization in the transportation sector (response rate = 70%) with truck drivers as the main employees of the organization. Only one branch of the company, situated in the center of Italy, was recruited for the research. The returned questionnaires were 208. Both companies were private and situated at the heart of Italy. The data collection was implemented by psychologists. Employees of both organizations were tested in their workplace during the working hours in the rooms provided by the organizations. No payment was provided to the participants. Prior to filling out the questionnaires, the participants were informed regarding the approximate time that it should take to complete all instruments; however, no time limit was imposed.

### Sample

A total of 346 employees returned the questionnaires. However, 20 questionnaires were deleted due to missing items, resulting in a final sample of 326 employees. Out of 326 respondents 80.9% were male and 19.1% were female. In detail, 259 employees were male and 56 were females, while 11 employees did not declare their gender. The sample included 24% white-collar employees and 76% blue-collar employees in operative jobs. Fifty-three percent of employees had a seniority of 7 years or less, whereas 47% had a seniority of more than 7 years (*M:* 1, 5, ds: 0.50). Because of the highly confidential nature of the study, it was agreed with the organizations that the information about age would not be collected. Bullying in Italy is a “hot” topic and caution is needed in its measurement as far as demographics are concerned (Giorgi et al., 2015c). Among the measured demographic characteristics, gender, and organizational tenure were included in our analyses in order to control for their effect on both the mediator and the dependent variables.

### Measures

#### Exposure to Workplace Bullying

It was measured using the Italian version of the *Negative Acts Questionnaire-Revised* (NAQ-R: Einarsen et al., 2009) validated by Giorgi et al. (2011). Participants indicated how frequently (e.g., 1: Never, 2: Now and then, 3: Monthly, 4: Weekly, and 5: Daily) they had been exposed to 17 specific bullying behaviors within the last 6 months (e.g., “being withheld information which affects your performance”). Both work-related and person-related bullying are measured by the NAQ-R.

#### Psychological Distress

It was measured using the 12-item Italian version of the *General Health Questionnaire* (GHQ: Goldberg, 1992). The GHQ is a self-administered screening instrument for psychiatric disorder in non-clinical populations that provides a more general measure of psychological well-being (e.g., “Feeling unhappy and depressed”). Dysphoria, anxiety and safeness are aspects of the psychological distress measure.

The responses were measured using a four-point Likert-type scale (0-1-2-3) and, after recoding some inverted items, we used the total score of the scale in the subsequent analyses.

#### Self-Management Ability

It was measured using the scale of self-management from the *Organizational Emotional intelligence Questionnaire* (ORG-EIQ, Giorgi and Majer, 2009). The scale of self-management included two competencies of emotional self-control (six items, e.g., “In the workplace I tend to be impulsive –reversed score-”) and tenacity (five items, e.g., “I don’t easily discourage in achieving my working goals”; Giorgi and Majer, 2009). Response ratings were measured on a five-point Likert-type scale. The internal consistency of the questionnaires was satisfactory, because it varied from 0.80 to 0.91 (see **Table 2**).

**Table 2:** Descriptive statistics and correlations.

Variables	*M*	*SD*	1	2	3	4	5
(1) Sex	–	–	–				
(2) Organizational tenure	–	–	0.14^∗^	–			
(3) Workplace bullying	22.06	5.27	0.08	0.12^∗^	(0.91)		
(4) Psychological distress	8.81	4.29	0.21^∗∗^	0.12^∗^	0.47^∗∗^	(0.83)	
(5) Ability of self-management	65.75	9.31	–0.15^∗∗^	–0.19^∗∗^	–0.25^∗∗^	–0.37^∗∗^	(0.80)

*N = 326. Internal consistency coefficients (Cronbach’s alphas) appear along the diagonal in parentheses. ^∗^p < 0.05, ^∗∗^p < 0.01.*

## Results

### Confirmatory Factor Analysis and Assessment of a Common Method Variance

Following Anderson and Gerbing’s (1988) two-step structural equation modeling (SEM) procedure, we tested a measurement model [confirmatory factor analysis (CFA)] by determining whether each measure’s estimated loading on its expected underlying factor was significant. This allowed us to establish discriminant validity among the study constructs. Then, a structural model was performed to estimate the fit of the hypothesized model to the data. A CFA was, therefore, conducted with the maximum likelihood estimation procedure with Mplus, version 7.11 (Muthén and Muthén, 1998-2010). The analysis was performed with the three variables measuring workplace bullying, psychological distress and self-management ability. Moreover, the variables’ dimensions were used as indicators of their corresponding latent constructs in the measurement and structural models. These dimensions were formed by averaging the items of each sub-scale for the three latent variables. We, therefore, obtained two indicators for workplace bullying, three indicators for psychological distress and two indicators for self-management.

To evaluate the model fit, we considered chi-square (the higher the values are, the worse is the model’s correspondence to the data), and used both absolute and incremental fit indexes. Absolute fit indexes evaluate how well an *a priori* model reproduces the sample data. In our study, we focused on the three absolute fit indexes: the standardized root mean square residual (SRMR), for which values of less than 0.08 are favorable, and the root-mean-square error of approximation (RMSEA), which should not exceed 0.10 (Browne and Cudeck, 1993; Kline, 2011). Incremental fit indexes measure the proportionate amount of improvement in fit when a target model is compared with a more restricted, nested baseline model (Schreiber et al., 2006). We considered the comparative fit index (CFI), for which values of 0.90 or greater are recommended (Schreiber et al., 2006). As expected, the hypothesized three-factor model yielded a good fit to the data: χ^2^(11) = 37.710, CFI = 0.95 RMSEA = 0.09; SRMR = 0.04. Additionally, as shown in **Table 2**, this model had a significantly better fit than alternative, more parsimonious models (*p* < 0.01), supporting the distinctiveness of the study variables (the standardized factor loadings are reported in the **Table A1**, Appendix).

However, since all the data were collected at the same time and included self-report scales, common method bias problems may arise and inflate the patterns of relationships among the study variables. Following the statistical recommendations of Podsakoff et al. (2003), we, thus, used the unmeasured latent method factor approach to control for the effects of common method variance. This approach was adopted because it does not require specifying the source of method bias, and it controls for any systematic variance among the items that are independent of the covariance because of the constructs of interest (Podsakoff et al., 2003). Therefore, this technique is particularly recommended when the specific source of the method bias is unknown or cannot be measured (Williams et al., 1989), as in our study. Accordingly, a common method factor was added to the hypothesized three-factor model to assess the potential increase in model fit that would be obtained from accounting for the unmeasured method factor. The model provided a better fit to the data than the same model without the method factor: χ^2^(4) = 2.841, CFI = 1.00, RMSEA = 0.00, SRMR = 0.01, Δχ^2^(3) = 34.869, *p* < 0.01. Nonetheless, the method factor accounted for 26% of total variance, which is not above the average portion of variance reported in self-report studies (Williams et al., 1989; Podsakoff et al., 2003). We can, therefore, conclude that a common method bias is unlikely to be a serious threat in our study.

All coefficients are significant at *p* < 0.01. Standard errors appear in parentheses.

### Hypothesis Testing

**Table 3** displays the descriptive statistics, correlations and reliability coefficients of the study variables. The significant negative relationship between exposure to workplace bullying and self-management ability supported Hypothesis 1.

**Table 3:** Fit indices for nested structural models.

Model	χ^2^	*df*	Δχ^2^	Δ*df*	CFI	RMSEA	SRMR
Model 1 (hypothesized fully mediated model)	51.175^∗^	22	–	–	0.94	0.06	0.04
Model 2 (partially mediated model)	51.173^∗^	21	0.002	1	0.94	0.07	0.04
Model 3 (non-mediated model)	63.342^∗^	23	12.167^∗^	2	0.92	0.07	0.06

*N = 326. CFI, comparative fit index; RMSEA, root-mean-square error of approximation; SRMR, standardized root mean square residual. ^∗^p < 0.01.*

In order to examine the hypothesized theoretical model, we performed SEM with Mplus. SEM offers the advantage of (a) controlling for measurement errors when the relationships among variables are analyzed (Hoyle and Smith, 1994), and (b) comparing the goodness-of-fit of the hypothesized model with other alternative models (Cheung and Lau, 2008). Hence, we tested our proposed structural model and compared it with alternative models.

**FIGURE 2:**
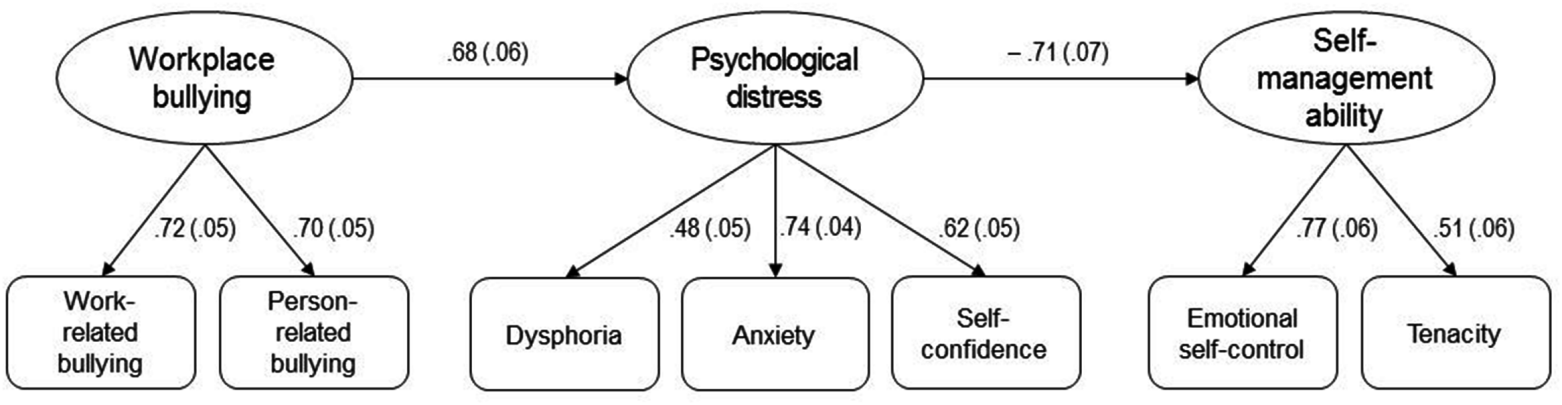
**Completely standardized path coefficients for Model 1. All coefficients are significant at *p* < 0.01. Standard errors appear in parentheses**.

The hypothesized model (Model 1), which is a fully mediated model, displayed a good fit to the data: χ^2^(22) = 51.175, CFI = 0.94; RMSEA = 0.06; SRMR = 0.04. Specific inspection of direct relationships further revealed that workplace bullying was positively associated with psychological distress (β = 0.68, *p* < 0.01), and that psychological distress, in turn, was negatively related to the ability of self-management (β = –0.71, *p* < 0.01). Completely standardized path coefficients for Model 1 are depicted in **Figure 2.**

To assess whether the hypothesized model was the best representation of the data, we then compared its fit to that of the two alternative models. First, we assessed a partially mediated model, which included an additional direct path from workplace bullying to self-management. This model yielded an adequate fit to the data (χ^2^[21] = 51.173, CFI = 0.94; RMSEA = 0.07; SRMR = 0.04), but it was not significantly better than Model 1, as revealed by the chi-square difference (Δχ^2^[1] = 0.002, *ns*). Moreover, the additional direct link between workplace bullying and self-management was not significant (β = 0.01, *ns*). Next, we compared the hypothesized model with a non-mediated model (Model 3), which only included the direct relationship between workplace bullying and psychological distress with self-management. Results revealed that the non-mediated model was a worse fit to the data than the hypothesized fully mediated model (χ^2^[23] = 63.342, CFI = 0.92; RMSEA = 0.07; SRMR = 0.06, Δχ^2^[1] = 12.167, *p* < 0.01).

Overall, results from the model comparison suggested that Model 1 was the best fitting and most parsimonious model. We, therefore, retained the hypothesized fully mediated model. In order to assess whether the indirect relationship between workplace bullying and self-management via psychological distress was significant (Hypothesis 2), we calculated 95% bootstrapping confidence intervals (Preacher and Hayes, 2008; Preacher and Kelley, 2011). Based on 5,000 bootstrap replications, results indicated that the indirect negative effect of workplace bullying on emotional intelligence via psychological distress was significant (indirect effect = –0.49; 95% CI = –0.59, –0.38). Hence, Hypothesis 2 was, therefore, supported.

Taken together, SEM results showed that workplace bullying was indirectly negatively associated with self-management ability via increased psychological distress, thereby lending empirical support for our theoretical model.

## Discussion

The aim of the present study was to investigate the relationship linking workplace bullying, psychological distress and the self-management ability of emotional intelligence. The results indicated that exposure to workplace bullying was linked to self-management ability and confirmed Hypothesis 1. One potential explanation for this relationship may be based on Ansbacher and Ansbacher (1956), who claimed that stressful circumstances at work (such as exposure to workplace bullying) may be perceived as a threat and lead an individual to active or passive self-defensive behaviors. As Dreikurs (1975) and Dreikurs (1996) also proposed, in stressful circumstances feelings of inferiority increase and, as a consequence, an individual becomes less aware of various choices for his/her reactions and behaviors. Hence, exposure to workplace bullying may impede flexibility of decision-making and increase the likelihood of impulsive behavior, which is linked to the lack of self-management ability.

Although emotional intelligence has been proved to be valuable in improving individual and organizational productivity and wellbeing (Cavallo and Brienza, 2004; Rosete and Ciarrochi, 2005; Yadav, 2014), only a few previous studies have addressed the question as to what contributes to the development or impediment of emotional intelligence. Workplace bullying as a potential threat has not been previously analyzed, although, a link between these two variables was established (e.g., Branch et al., 2012). For example, Branch et al. (2012) demonstrated that there is a relationship between bullying and emotional intelligence and Ashraf and Khan (2014) showed that emotional intelligence moderated the relationship between workplace bullying and job performance. Sheehan (1999) proposed that, for the prevention of workplace bullying, the developing of employees’ emotional intelligence may be useful. Our theoretical model and results of the present study suggest that the link between workplace bullying and emotional intelligence may be the other way around, i.e., workplace bullying may impede the development of emotional intelligence (more specifically, the development of the self-management ability).

Our results also demonstrate that workers who perceived greater exposure to workplace bullying report greater levels of psychological distress. These findings are consistent with previous empirical findings, demonstrating that individuals, experiencing workplace bullying face serious psychological consequences (Nielsen and Einarsen, 2012; Nielsen et al., 2012).

Finally, the present data analysis revealed that there is an indirect relationship between exposure to workplace bullying and self-management ability, fully explained by psychological distress. Hence, it seems that suffering from mental health problems (such as dysphoria, anxiety and feelings of insecurity) that arise due to exposure to workplace bullying, weaken the emotional ability of self-management. Previous studies have shown that psychological distress is linked to impulsive and risky behaviors that contradict the self-management ability, e.g., risky sexual behavior and substance use (Elkington et al., 2010). In addition, empirical findings have demonstrated that there is a link between general emotional intelligence and psychological distress (Karim, 2009). On the other hand, no studies have documented the potential impediment of emotional intelligence considering exposure to workplace bullying and psychological distress. Thus, overall, it seems that not only is emotional intelligence fundamental for handling stressful events and relationships, but also a non-bullying environment is important in developing employees’ self-management ability.

The present study contributes to the research field of workplace bullying and emotional intelligence in several ways. First, the hypothesis that emotional resources are associated with exposure to workplace bullying has been explored in different studies, but research has not tested whether emotional intelligence may deplete as a consequence of exposure to workplace bullying. Second, in the present study, we aimed at testing and expanding the Cherniss and Goleman (2001) model of emotional intelligence and organizational effectiveness by including psychological distress as a construct explaining the link proposed in the model between relationship and emotional intelligence. Third, we believe that our model may be a new avenue to consider for applied use in the workplace bullying field, suggesting that controlling for the incidences of workplace bullying is critical for employees’ emotional intelligence and, consequently, organizational and individual success.

### Limitations

The cross-sectional nature of the study precludes any causal conclusion regarding the present study. The mediation effect explored might be biased due to the lack of a longitudinal design. Although we constructed our hypotheses on theoretical arguments and previous empirical research, the cross-sectional nature of the present study limits the accuracy of our findings. This challenge is particularly relevant in the case of mediation analysis, since methodologists have shown that cross-sectional models of mediation are biased relative to the expected causal processes, and that the bias can occur in either direction, depending on the structure of the supposed causal model (Maxwell and Cole, 2007; Maxwell et al., 2011). In addition, Cherniss and Goleman (2001) proposed that not only may interpersonal relationships affect emotional intelligence, but there could also be a reciprocal relationship. Hence, longitudinal research needs to be implemented to test for the reverse relationship between emotional intelligence and workplace bullying over time.

In the present study, we considered only one mediating variable; therefore, future studies may wish to extend this model by considering other potential mechanisms explaining the indirect relationship. In line with that, Cherniss and Goleman (2001) also claimed that interventions that focus on only one part of the emotional intelligence and organizational effectiveness model are less likely to be effective. Hence, future research should address the full model, as this will better ensure practical use of the study findings.

In the future, it is important to analyze all four abilities of emotional intelligence (self-awareness, self-management, social awareness, relationship management), because, as Goleman, (2001, p. 39) argued, “people exhibit competencies in groupings, often across clusters that allow competencies to support one another”.

The higher proportion of men (80.9%) in our study means that further studies should be implemented in female-oriented organizations. Indeed, gender differences seem to be important to consider in emotional intelligence investigations (Khalili, 2011; Naghavi and Redzuan, 2011).

In future studies, it may be important to analyze individual differences. As Balducci et al. (2009) proposed, individuals characterized by the neurotic triad on Minnesota Multiphasic Personality Inventory (MMPI) tend to manifest, especially when under stress, and are more prone to implement dysfunctional defensive mechanisms such as somatization, denial, and repression. Hence, it could be that, among certain individuals, a depletion of the abilities of emotional intelligence may be more pronounced.

## Author Contributions

GG, MP, FM, JF-P, NM, and GA equally contributed to all the following issues of the research: conception and design of the work; acquisition, analysis, or interpretation of data for the work; drafting the work and critically revising it; final approval of the version to be published; agreement to be accountable for all aspects of the work in ensuring that questions related to the accuracy or integrity of any part of the work are appropriately investigated and resolved.

## Conflict of Interest Statement

The authors declare that the research was conducted in the absence of any commercial or financial relationships that could be construed as a potential conflict of interest.

## APPENDIX

**TABLE A1:** Completely Standardized Factor Loadings for the Study Variables.

Factor/item	Estimate
**Workplace bullying**	
Work-related bullying	0.72
Person-related bullying	0.70
**Psychological distress**	
Dysphoria	0.48
Anxiety	0.74
Loss of confidence	0.62
**Self-management**	
Emotional self-control	0.77
Tenacity	0.51

*N = 326. All factor loadings were significant at p < 0.01.*
